# Sinus venous thrombosis as a complication of COVID-19-associated hypercoagulability

**DOI:** 10.1186/s41983-021-00387-0

**Published:** 2021-09-26

**Authors:** Sinda Zarrouk, Josef Finsterer

**Affiliations:** 1 University of Tunis El Manar and Genomics Platform, Pasteur Institute of Tunis, Tunis, Tunisia; 2 Klinik Landstrasse, Messerli Institute, Postfach 20, 1180 Vienna, Austria

**Keywords:** SARS-CoV-2, COVID-19, Hypercoagulability, Thrombosis, Complications

## Abstract

Sinus venous thrombosis (SVT) is an increasingly recognised complication of not only SARS-CoV-2 infections, but also of SARS-CoV-2 vaccinations. SVT is attributed to hypercoagulability, a common complication of COVID-19, disregarding the severity of the infection. Hypercoagulability in COVID-19 is explained by direct activation of platelets, enhancing coagulation, by direct infection and indirect activation of endothelial cells by SARS-CoV-2, shifting endothelial cells from an anti-thrombotic to a pro-thrombotic state, by direct activation of complement pathways, promoting thrombin generation, or by immune thrombocytopenia, which also generates a thrombogenic state. Since SVT may occur even in anticoagulated COVID-19 patients and may have an unfavourable outcome, all efforts must be made to prevent this complication or to treat it accurately.

## Introduction

With interest we read the article by Sittanggang and colleagues about two patients (58-year-old female (patient-1), 72-year-old male (patient-2)) experiencing sinus venous thrombosis (SVT) during a SARS-CoV-2 infection [1]. Latency between the onset of COVID-19 and the onset of SVT was 7 and 17 days in patient-1/patient-2, respectively [1]. The onset manifestations were seizure and hemiparesis (patient-1) and impaired consciousness (patient-2), respectively [1]. It was concluded that COVID-19 can be complicated by SVT, that the prognosis of SVT is poor if drainage of the brainstem is compromised, that hypercoagulability is a complication of COVID-19 if arterial hypertension and diabetes are present, and that coagulation cascades are prolonged despite normal D-dimer [1]. The study is appealing, but raises comments and concerns.

## Main text

We do not agree with the statement that hypercoagulability is a complication of COVID-19 only if there is concomitant arterial hypertension or diabetes [1]. Hypercoagulability is a common complication not only of severe, but also of mild or moderate COVID-19 [2] and explained by (1) direct activation of platelets, enhancing coagulation; (2) direct infection and indirect activation of endothelial cells by the cytokine storm, shifting endothelium from an anti-thrombotic to a pro-thrombotic state, and (3) direct activation of complement pathways, promoting thrombin generation [3]. Since SARS-CoV-2 activates the immune system, autoimmune disease is a frequent complication [4], including immune thrombocytopenia, which may further enhance the thrombogenic state in COVID-19 patients [5]. There are also indications for alterations of the clotting factor titers and activation of platelets, which leads to thrombus formation in coronary arteries and cerebral arteries [6].

A limitation of the study is that only computed tomography (CT) scans of the brain were provided. Since SVT is frequently complicated by ischaemic stroke with or without consecutive bleeding [7], and since ischaemic stroke, particularly small ones, are only detectable on multimodal magnetic resonance imaging (MRI) [8], it is worthwhile to know if multimodal MRIs of the brain are available and if ischaemic stroke was documented on MRI in either patient. Since SVT may be associated also with bleeding, we should know if cerebral CT scans demonstrated intracerebral bleeding in either patient.

The statement of the conclusions “upon monitoring the neutrophil/lymphocyte ratio, it became apparent that D-dimer, fibrinogen, and C-reactive protein (CRP) are bad prognosis indicators, why the use of heparin or low molecular weight heparin (LMWH) decreases the D-dimer and not by any means increase the symptoms” [1] is inconclusive. Do the authors mean that LMWH decreases the D-dimer levels but not the symptoms, or do they mean that low D-dimer is associated with reduction of symptoms? This should be clarified. Recently, it has been proposed to apply standardised viscoelastic tests, such as TEG, Quantra, ROTEM, or ClotPro, to assess haemostasis and the thrombotic risk in COVID-19 patients [9].

Patient-1 was described as having arterial hypertension as the only peculiarity in her previous history. However, according to table 1 in the article by Sittanggang and colleagues the HbA1c value was 6.5, suggesting that the patient was also diabetic. We should know if diabetes was first diagnosed during hospitalisation or previously known.

Missing is the drug history. We should be told if either patient was regularly taking hormones. Missing is an extensive history for other risk factors for SVT, such as smoking, antiphospholipid syndrome, thrombophilia (elevated protein-S or protein-C), or inflammatory bowel disease [10].

Table 1 of the article by Sittnaggang and colleagues does not provide reference limits of any parameter listed. This is why it cannot be assessed which of the listed figures of table 1 are normal or abnormal. It is also unclear what is meant with “INR 5.308 (before 50,000) (patient-2)” It should be mentioned if hemiparesis in patient-1 was right-sided or left-sided.

## Conclusions

The presented study has limitations, which weaken the findings and their interpretation. These limitations should be addressed to further strengthen the conclusions.

## Data Availability

All data are available from the corresponding author.

## Abbreviations

CRP: C-reactive protein
CT: Computed tomography
LMWH: Low molecular weight heparin
MRI: Magnetic resonance imaging
SVT: Sinus venous thrombosis
